# Current progress in antibacterial agents for carbapenem-resistant enterobacterales

**DOI:** 10.3389/fphar.2026.1796557

**Published:** 2026-04-01

**Authors:** Wenxin Xu, Lisha Shang, Xue Li, Jie Yu, Jinyi Shen, Yujin Li, Qiongyao He, Liang Chen, Deqiang Wang, Yelin Wei, Siqiang Niu

**Affiliations:** 1 Department of Laboratory Medicine, The First Affiliated Hospital of Chongqing Medical University, Chongqing, China; 2 College of Laboratory Medicine, Chongqing Medical University, Chongqing, China; 3 Department of Pharmacy Practice, School of Pharmacy and Pharmaceutical Sciences, University at Buffalo, Buffalo, NY, United States; 4 The Key Laboratory of Molecular Biology of Infectious Diseases designated by the Chinese Ministry of Education, Chongqing Medical University, Chongqing, China; 5 Department of Laboratory Medicine, The First people’s Hospital of Xiaoshan Hangzhou, Hangzhou, Zhejiang, China

**Keywords:** antimicrobial agents, carbapenem-resistant enterobacterales, combinationtherapy, cre, resistance mechanisms

## Abstract

*Carbapenem-resistant Enterobacterales* (CRE) have been identified by the World Health Organization as critical-priority pathogens, posing a severe global public health threat due to limited therapeutic options and high mortality rates. In response, this review provides a comprehensive assessment of current antimicrobial strategies against CRE, covering the efficacy and limitations of traditional antibiotics (e.g., polymyxins, tigecycline), novel agents (e.g., cefiderocol, ceftazidime-avibactam), and diverse combination therapies. Despite recent advances, major gaps persist, including the lack of effective regimens for pan-drug-resistant (PDR) strains, insufficient large-scale clinical evidence for many novel agents, and disparities in global access to newer therapeutics. Future priorities should including conducting high-quality randomized controlled trials (RCTs) to optimize treatment strategies, integrating rapid molecular dianostics into routine clinical practice to facilitate precision therapy, and continuing the development of novel agents and synergistic combination approaches. Simultaneously, establishing a global antimicrobial resistance surveillance network is indispensable for mitigating the ongoing spread of CRE.

## 1 Introduction

CRE are generally defined as Enterobacterales that are resistant to at least one carbapenem antibiotic (2025a)^1^. In recognition of its serious public health impact, CRE was listed as a critical priority pathogen in the 2024 WHO priority pathogens list, owing largely to limited therapeutic options and continuously escalating resistance trends (Sati et al., 2025). Since the first being documented in the early 1990s (Lee et al., 1991), CRE rapidly disseminated to various countries in the 21st century and is now considered a pervasive threat in healthcare settings worldwide. The clinical burden of CRE infections remains substantial, as evidenced by an international cohort study in which even with appropriate treatment, the 30-day all-cause mortality rate for CRE BSIs reached 38.5% (Gutierrez-Gutierrez et al., 2017).

CRE are frequently associated with resistance to non-β-lactam antibiotics, which often — but not invariably — results in multidrug-, extensively drug-, or pandrug-resistant phenotypes, resulting in limited therapeutic options. Concurrently, CRE infections are linked to increased mortality rates, prolonged hospital stays, and elevated healthcare costs (Tompkins and van Duin, 2021). Immunocompromised patients constitute the high-risk population for CRE infections, including those with hematologic malignancies and solid organ transplant recipients, among whom infection is associated with substantial mortality (Pouch and Satlin, 2017; Herrera et al., 2024). In healthcare settings, CRE infections are primarily observed among severely ill patients (Fochat et al., 2024), and these pathogens also pose a potential risk of community transmission (Kock et al., 2018; Goncalves et al., 2024).

Given the persistent global spread of CRE and its severe clinical impact, a contemporary and critical synthesis of available and emerging treatment strategies is urgently needed. This review maps the current antimicrobial landscape for CRE by analyzing literature retrieved from PubMed between 1983 and 2025. We not only summarize established agents but also interpret the latest evidence on novel therapies and combination regimens. Ultimately, this work aims to provide a comprehensive, actionable knowledge framework to inform clinical medication use.

## 2 Mechanisms of drug resistance

Primary resistance mechanisms in CRE include the production of carbapenemases, loss or alteration of outer membrane porins, overexpression of efflux pumps, and modification of penicillin-binding proteins (PBPs) (Logan and Weinstein, 2017). Among these, the production of carbapenemases is the predominant mechanism (Logan and Weinstein, 2017; Ma et al., 2023).

### 2.1 Carbapenemase-mediated resistance

Carbapenemase genes are frequently harbored by transferable plasmids (e.g., IncF and IncN types) and can transmit among bacterial species through horizontal gene transfer (Kopotsa et al., 2019). The Ambler molecular classification system categorizes carbapenemases into three classes: A, B, and D. *Klebsiella pneumoniae* carbapenemase (KPC), the most prevalent type among Class A enzyme, predominates in *K. pneumoniae* (Ma et al., 2023). Class B enzymes, designated as metallo-β-lactamases (MBLs), include imipenemase (IMP), Verona integron-encoded metallo-β-lactamase (VIM), and *New Delhi* metallo-β-lactamase (NDM) types, among others. Oxacillinase-48 carbapenemase (OXA-48) and OXA-48-like carbapenemases, one of Class D serine carbapenemase, is commonly identified in *K. pneumoniae* and *Escherichia coli* (Mairi et al., 2018).

### 2.2 Non-enzymatic resistance mechanisms

Non-enzymatic resistance mechanisms encompass altered membrane permeability, enhanced efflux pump activity, and modification of target proteins. In *K. pneumoniae*, deletion or mutation of OmpK35/OmpK36 porins (e.g., in the ST11 clone lineage) significantly impairs the intracellular penetration of carbapenems, thereby reducing membrane permeability (Potter et al., 2016). Overexpression of the AcrAB-TolC system leads to heightened efflux pump activity, actively expelling drugs to lower intracellular concentrations and synergistically enhancing resistance with enzymatic mechanisms (Potter et al., 2016). Although some studies have reported reduced affinitty of carbapenems to modified PBPs, this mechanism remains infrequently observed in Enterobacterales (Zapun et al., 2008).

## 3 Current antimicrobial agents

### 3.1 First-line therapy for severe CRE infections

#### 3.1.1 Cefiderocol

Cefiderocol, a novel broad-spectrum cephalosporin antibiotic, combines the iron-carrier activity of siderophores with the antibacterial properties of β-lactams to enhance its penetration through the outer membrane of Gram-negative bacteria and reduce the susceptibility to β-lactamases (Ito et al., 2018). Within bacterial cell, cefiderocol specifically binds to PBP3 to inhibit cell wall synthesis and exert bactericidal effects (Smith et al., 2024).

The efficacy of cefiderocol has been validated in both *in vitro* studies and clinical trials. At the molecular and strain levels, cefiderocol exhibits broad-spectrum anti-CRE activity. A study including 603 clinical CRE isolates reported an overall susceptibility rate of 92% to cefiderocol, with 94% susceptibility among KPC-producing strains and 83% in NDM-producing strains (Tamma et al., 2023b). In a Spanish study involving 57 dual-carbapenemase-producing isolates, cefiderocol also showed high susceptibility (53/57) (Blanco-Martin et al., 2024). Notably, cefiderocol’s activity is subject to the inoculum effect: in high bacterial load infections (such as severe pneumonia or sepsis), minimum inhibitory concentration (MIC) values may increase. This phenomenon has been observed in about 90% of cefiderocol-susceptible CRE strains, suggesting a potential risk of monotherapy failure in severe infections and highlighting the need for combination regimens (Huang et al., 2023). In pivotal clinical trials, cefiderocol showed similar efficacy comparable to that of the best available therapy (BAT). In the APEKS-cUTI trial, cefiderocol was non-inferior to imipenem-cilastatin (73% vs. 55%) (Portsmouth et al., 2018). The CREDIBLE-CR trial (targeting CRE-caused complicated urinary tract infections (UTIs), hospital-acquired pneumonia (HAP), and sepsis) demonstrated that the cefiderocol group had similar clinical cure rates and microbiological eradication rates compared to BAT (pre-specified by the investigator before randomisation and comprised of a maximum of three drugs) (Bassetti et al., 2021). In the APEKS-NP trial, cefiderocol demonstrated non-inferiority to high-dose, extended-infusion meropenem for Gram-negative nosocomial pneumonia, with comparable efficacy observed against infections caused by carbapenem-resistant pathogens (Wunderink et al., 2021).

Cefiderocol is effective against multiple CRE phenotypes, and has been approved by the Food and Drug Administration (FDA) for the treatment of complicated UTIs in adults. The European Medicines Agency (EMA) also recognizes its therapeutic potential against CRE infections. Clinically, it is often considered as a last-resort therapeutic option, particularly for multidrug-resistance (MDR) infections (Aslan and Akova, 2024). Future efforts should include expanding its approved indications, optimizing combination treatment strategies, and enhancing its utility in clinical CRE therapy.

#### 3.1.2 Ceftazidime-avibactam

Ceftazidime-avibactam is a combination agent with potent activity against CRE. Ceftazidime exhibits broad-spectrum activity against Gram-negative bacteria but is susceptible to hydrolysis by β-lactamases (Vougiouklakis et al., 2025). Avibactam, as a novel β-lactamase inhibitor, restores the antibacterial activity of ceftazidime by reversibly inhibiting Ambler class A, class C, and some class D β-lactamases, including extended-spectrum β-lactamases (ESBLs), AmpC, and KPC enzymes, but it is ineffective against MBLs-producing strains (Aktas et al., 2012).

Ceftazidime-avibactam has demonstrated favorable efficacy in the treatment of CRE infections. A systematic review based on the included RCTs demonstrated that ceftazidime-avibactam had non-inferior efficacy to carbapenems for treating Enterobacterales infections, with comparable clinical success rates (Sternbach et al., 2018). The CAVICOR study further confirmed that ceftazidime-avibactam serves as an effective alternative treatment regimen, particularly in high-risk patients with INCREMENT-CRE score of >7 points (Caston et al., 2022). A clinical study on carbapenem-resistant *K. pneumoniae* (CRKP) found that replacing colistin with ceftazidime-avibactam reduced mortality (9% vs. 32%) (van Duin et al., 2018). For the treatment of bacteremia caused by KPC-producing CRE, ceftazidime-avibactam demonstrates superior clinical efficacy and a more favorable safety profile, including lower rates of nephrotoxicity, in comparison to traditional regimens that incorporate aminoglycosides or colistin (Shields et al., 2017). A retrospective cohort study showed similar clinical success rates between ceftazidime-avibactam and meropenem-vaborbactam for CRE infections, but the incidence of resistance with ceftazidime-avibactam may be higher (Ackley et al., 2020). Monotherapy with ceftazidime-avibactam can lead to resistance development, primarily through KPC gene variants or transfer (such as blaKPC-3 mutations) (Gottig et al., 2019). *In vitro* studies comfirm that KPC-producing strains can rapidly develop resistance, and avibactam may drive KPC evolution, leading to enhanced ceftazidime hydrolysis and increased resistance occurrence (Wei et al., 2025).

Research has shown that although ceftazidime-avibactam monotherapy offers a broad antibacterial spectrum and favorable safety profile, it is prone to resistance development. In contrast, combining ceftazidime-avibactam with other antibiotics not only enhances therapeutic efficacy but also significantly reduces the likelihood of resistance emergence. Consequently, the use of ceftazidime-avibactam in combination regimens is recommended. Synergistic effects have been observed in an *in vitro* study of CRKP when ceftazidime-avibactam is combined with colistin and fosfomycin (Tuzemen et al., 2024), and its combination with imipenem has been shown suppress the emergence of resistant subpopulations (Gottig et al., 2019). The combination of ceftazidime-avibactam and aztreonam demonstrates favorable efficacy against MBLs-producing CRE. In a study by Falcone et al. focusing on BSIs, the 30-day mortality rate was 19.2% for patients receiving the ceftazidime-avibactam-aztreonam regimen, compared to 44% in those receiving alternative therapies (Falcone et al., 2021). Recent research by Huespe et al. further supports this, confirming that the combination was associated with lower mortality (35% vs. 47%) and reduced clinical failure rates (46% vs. 53%) compared to other alternative regimens (Huespe et al., 2025).These findings collectively suggest the potential of ceftazidime-avibactam-based combination therapies in the management of CRE infections.

Ceftazidime-avibactam is approved for the treatment of complicated UTIs, complicated intra-abdominal infections (IAIs), HAP and ventilator-associated pneumonia (VAP). Common adverse effects include nausea and headache (Shirley, 2018). Ceftazidime-avibactam is regarded as a first-line treatment for OXA-48-like-producing strains and an alternative option for KPC-producing strains, but it is ineffective against MBLs-producing strains. Compared with carbapenems, ceftazidime-avibactam has been associated with a higher incidence of serious adverse events, including nephrotoxicity and allergic reactions (Sternbach et al., 2018). In summary, ceftazidime-avibactam is a valuable tool against MDR pathogens, but judicious clinical use is essential to minimize resistance development and maintain long-term efficacy.

#### 3.1.3 Meropenem-vaborbactam

Meropenem-vaborbactam consists of the carbapenem antibiotic meropenem and the novel β-lactamase inhibitor vaborbactam. Vaborbactam specifically inhibits class A serine-β-lactamases (SBLs), blocking their hydrolysis of meropenem and thereby restoring its antibacterial activity (Dhillon, 2018).

Meropenem-vaborbactam shows strong antibacterial activity against CRE in both *in vitro* and clinical trials. *In vitro* activity tests found that this combination has the strongest inhibitory effect on KPC-producing CRE. A U.S. study involving 1,697 MDR Enterobacterales isolates showed that meropenem-vaborbactam had an inhibition rate of 99.1%, including 98.9% inhibition among 222 KPC-producing CRE isolates (Shortridge et al., 2023). However, a European *in vitro* study also found that it showed low activity against class B MBL-producing strains (1.7%) and moderate activity against class D OXA-48-like producers (40.5%) (Shortridge et al., 2021). In the TANGO II randomized clinical trial, meropenem-vaborbactam demonstrated significantly higher clinical cure rates and a more favorable safety profile than BAT (mono/combination therapy: polymyxins, carbapenems, aminoglycosides, tigecycline; or ceftazidime/avibactam alone) in the treatment of serious CRE infections (Wunderink et al., 2018). Meropenem-vaborbactam is generally well-tolerated. Common adverse reactions include headache and injection-site reactions. It exhibits mild hepatotoxicity, with a slightly increased alanine aminotransferase levels, although these elevations are typically transient and reversible (Gibson, 2019).

Meropenem-vaborbactam has been approved by the FDA for the treatment of complicated UTIs, including pyelonephritis (FDA, 2017). The EMA has expanded its indications to include complicated UTIs (including pyelonephritis), complicated (European Medicines Agency, 2018). The combination demonstrates excellent efficacy against KPC-producing CRE, particularly in complicated UTIs and pyelonephritis. However, its lack of activity against MBLs and OXA-48 limits its clinical applicability, underscoring the necessity for precise antimicrobial susceptibility testing prior to use.

#### 3.1.4 Aztreonam-avibactam

Aztreonam-avibactam is designed to treat MDR Gram-negative bacteria. Aztreonam can evade hydrolysis by MBLs but is sensitive to ESBLs and AmpC enzymes (Al Musawa et al., 2024). Avibactam effectively inhibits SBLs, but has no inhibitory effect against MBLs (Abboud et al., 2016). The combination of aztreonam with avibactam enables antibacterial activity against all β-lactamase-producing CRE.

*In vitro* and clinical studies have shown that aztreonam-avibactam has significant bactericidal activity against CRE. In a global surveillance study, aztreonam-avibactam inhibited 99.6% of CRE isolates at concentrations of ≤8 mg/L (MIC_50/90_, 0.25/0.5 mg/L) (Sader et al., 2024a). Studies found that the susceptibility rate of aztreonam-avibactam to CRE (87.8%–99.9%) was higher than that of ceftazidime-avibactam (81.6%), meropenem-vaborbactam (65.3%), and cefditoren (87.8%) (Sader et al., 2024b). A study of Chinese NDM-producing Enterobacterales strains reported that aztreonam-avibactam exhibited antibacterial activity against 99% of strains (Liu X. et al., 2025). In the Phase III REVISIT trial involving 422 patients with serious Enterobacterales infections, aztreonam-avibactam demonstrated non-inferiority to meropenem in clinical cure (68.4% vs. 65.7%) and exhibited a more favorable 28-day all-cause mortality profile (4% vs. 7%) (Carmeli et al., 2025). Meanwhile, *in vitro* studies have also revealed that combination therapy may enhance the antibacterial activity of aztreonam-avibactam. For instance, the combination of auranofin with aztreonam-avibactam significantly reduced the MIC against MBL-producing Enterobacterales (Wang et al., 2021).

Aztreonam-avibactam has been approved for the treatment of complicated IAIs in adults caused by Gram-negative bacteria. The most common adverse events include transient elevations in liver enzymes (9/34, 26.5%) and diarrhea (5/34, 14.7%) (Cornely et al., 2020). Though aztreonam-avibactam has become a core therapeutic option against MBLs-producing strains, future clinical practice should incorporate optimized dosing strategies based on antimicrobial resistance surveillance and consider combination regimens to broaden its therapeutic applications.

#### 3.1.5 Imipenem-cilastatin-relebactam

Imipenem-cilastatin-relebactam is a novel β-lactam/β-lactamase inhibitor combination. Its activity is largely attributable to relebactam, which targets Ambler class A and class C β-lactamases, including clinically important carbapenemases such as KPC (Smith et al., 2020). Although lacking direct antibacterial activity, relebactam restores imipenem’s activity against carbapenem-non-susceptible Enterobacterales and *Pseudomonas aeruginosa* by irreversibly inhibiting β-lactamases (Hilbert et al., 2023). However, as relebactam cannot inhibit MBLs activity, this combination is ineffective against MBL-producing strains (Haidar et al., 2017).

Imipenem-cilastatin-relebactam has demonstrated significant efficacy in both *in vitro* and clinical studies, especially in CRE infections. *In vitro* activity studies show that this combination has high susceptibility against most non-MBL-producing CRE. One study reported that relebactam increased imipenem susceptibility from 8% to 88% among CRE isolates (Haidar et al., 2017). An evaluation of 401 clinical CRE isolates in Spain showed that this combination effectively restored imipenem activity, particularly against KPC-producing and ESBL-producing strains (Vazquez-Ucha et al., 2021). In the phase III RESTORE-IMI 1 trial, it demonstrated comparable efficacy to imipenem + colistin in treating complicated UTIs and complicated IAIs, showing effectiveness against imipenem-non-susceptible Gram-negative infections (Smith et al., 2020). Another study supports its effectiveness in pneumonia-related infections, such as HAP and VAP (Sellares-Nadal et al., 2021). Imipenem-cilastatin-relebactam exhibits favorable safety and tolerability. Large trials (e.g., RESTORE-IMI 1) reported that the adverse event rate of the combination was comparable to imipenem + colistin, with no major toxicity or hypersensitivity reactions reported. Its safety is partly attributed to cilastatin, which prevents renal dehydropeptidase-mediated degradation of imipenem, while relebactam has not been associated with significant safety concerns (Smith et al., 2020).

This agent is currently approved by the FDA for the treatment of complicated UTIs and complicated (FDA, 2019). In several regions, it has been incorporated into guidelines as a therapeutic option for CRE (Smith et al., 2020; Heo, 2021). Future attention should be paid to monitoring emerging resistance patterns and expanding clinical indications through additional trials to secure its role in global antimicrobial stewardship.

### 3.2 Older agents recommended in guidelines

#### 3.2.1 Fosfomycin

As an epoxide antibacterial agent, fosfomycin inhibits the initial stages of bacterial cell wall synthesis through specific inhibition of the MurA enzyme (Falagas et al., 2019). This unique mechanism allows fosfomycin to achieve high intracellular concentrations and maintain activity against various MDR strains, including ESBLs and carbapenemase-producing isolates (Reffert and Smith, 2014). In an *in vitro* study involving 8 clinical CRE isolates, fosfomycin exhibited potent antimicrobial activity, with MIC values ranging from 0.5 to 64 mg/L (Lim et al., 2020).

Currently, fosfomycin is widely used in the treatment of CRE infections, with both oral and intravenous administrations. Oral fosfomycin is recommended for uncomplicated UTIs and remains effective against common pathogens (Michalopoulos et al., 2011). Intravenous fosfomycin is typically reserved for serious infections, including CRE-associated UTIs, bacteremia, and pneumonia (Shorr et al., 2017). A clinical study involving 94 patients demonstrated an overall clinical efficacy rate of 75.3%, with the highest response rate (92%) noted in UTIs (Aysert-Yildiz et al., 2023). A key limitation is its potential to select for emergent resistance during treatment, notably via *fosA* expression in *K. pneumoniae* (Ghayaz et al., 2023). Furthermore, fosfomycin in combination with carbapenems or quinolones significantly augments bactericidal activity against CRE by suppressing bacterial regrowth, particularly in UTIs (Loose et al., 2019; Lim et al., 2020).

Fosfomycin is currently approved for the treatment of uncomplicated UTIs, primarily against those caused by KPC-producing CRE. Intravenous fosfomycin has also received FDA approval for the treatment of complicated FDA (2025). Although generally well-tolerated, its most frequently reported adverse effect is gastrointestinal disturbance (Reffert and Smith, 2014). Given rising resistance, high-quality trials are urgently needed to optimize dosing and combination strategies.

#### 3.2.2 Polymyxins

Polymyxins, primarily comprising polymyxin B and colistin, exhibit potent efficacy against MDR Gram-negative bacteria, including *K. pneumoniae*, *Acinetobacter baumannii*, and *P*. *aeruginosa*. Clinical studies have demonstrated rapid bactericidal effects of polymyxin B *in vitro*, affirming its status as a last-line therapeutic agent for the clinical management of MDR and PDR infections (Zavascki et al., 2007).

In the management of CRE infections, polymyxins are administered as monotherapy or in combination regimens, with combination therapy generally demonstrating superior outcomes. Polymyxin B monotherapy is linked to relatively higher mortality rates. In a study focusing on CRKP BSIs, a polymyxin B-based regimen (as part of combination therapy) was associated with a 30-day mortality rate of 52.5% (21/40), indicating significant risk (Liang et al., 2019). In a separate study on CRKP pneumonia, the 28-day all-cause mortality reached 75.86% (22/29) in the standard-dose polymyxin B group, which was significantly higher than the 45.45% (20/44) observed in the tigecycline group (Chen et al., 2024). Given the increasing rates of resistance to polymyxins (Thomsen et al., 2023), they are frequently combined with other antibiotics to enhance therapeutic efficacy. Time-kill curve studies have confirmed that polymyxin B in combination with tigecycline or meropenem shows synergistic antibacterial effects against CRE, and polymyxin B plus meropenem regimen can also suppress bacterial regrowth (Alves et al., 2019). In a study of 96 clinical CRE isolates, the combination of colistin and mefloquine enhanced colistin susceptibility in 98.9% (95/96) of isolates, accompanied by marked reductions in MIC values (Zou et al., 2025). Another study demonstrated that zidovudine can reduce the MIC of polymyxin against *K*. *pneumoniae* by approximately 5-fold (Kaur et al., 2025). Additionally, a study has also indicated that the triple combination therapy with polymyxin, rifampin, and amikacin exhibited significantly enhanced bactericidal activity compared to dual combinations (Aye et al., 2020). These findings provide valuable insights of polymyxin-based combination therapies for the treatment of CRE infections.

Despite demonstrating high *in vitro* activity against multiple CRE phenotypes, polymyxins are primarily reserved as a last-resort therapeutic option because of nephrotoxicity and high mortality (Chen et al., 2024). Clinical practice is shifting toward combination therapies to enhance therapeutic efficacy and mitigate resistance (Zhang et al., 2017).

#### 3.2.3 Aminoglycosides

Aminoglycosides irreversibly bind to the bacterial 30S ribosomal subunit, disrupting protein synthesis and subsequently leading to bacterial death (Mehta and Champney, 2002). As carbapenemases do not directly impair aminoglycoside activity, these agents may still represent a viable therapeutic option in cases where CRE exhibit resistance to β-lactams (Zavascki et al., 2017).

Clinical evidence regarding aminoglycoside monotherapy for CRE infections remains limited. One study showed that CRE strains are more susceptible to amikacin than gentamicin (60.7% vs. 33%), while NDM-positive isolates exhibit higher aminoglycoside susceptibility compared with KPC-positive isolates (gentamicin: 46.8% vs. 7.2%; amikacin: 87% vs. 14.5%) (Lin et al., 2021). Other studies have found that aminoglycosides can enhance the permeability of the Gram-negative bacterial outer membrane, thereby augmenting the bactericidal effects of other antibiotics such as carbapenems and tigecycline (Yadav et al., 2015; Ma et al., 2022). This highlights the therapeutic potential of aminoglycoside-based combination therapies for the treatment of CRE infections.

Plazomicin, a novel aminoglycoside antibiotic, evades degradation by aminoglycoside-modifying enzymes through structural modification and exhibits superior *in vitro* activity against certain CRE strains compared to traditional aminoglycosides (Yoo, 2018). However, its activity is limited against OXA-48- and NDM-producing strains (Oztas et al., 2024). Clinically, plazomicin has demonstrated superior efficacy to meropenem in treating cUTIs (Eljaaly et al., 2019). Furthermore, plazomicin-based combination regimens are associated with lower mortality and fewer adverse events than colistin-based regimens (Eljaaly et al., 2019). Approved by both the FDA and EMA for cUTIs, plazomicin represents a valuable new therapeutic option for CRE infections.

#### 3.2.4 Tigecycline

Tigecycline, a semi-synthetic glycylcycline antibiotic, is widely used in the treatment of CRE infections by binding to the bacterial 30S ribosomal subunit to inhibit protein synthesis (Mehta and Champney, 2002).

Tigecycline exhibits high *in vitro* bacteriostatic activity against CRE, though its effectiveness is influenced by bacterial strain phenotype, MIC values, and treatment regimens. Notably, CRE isolates generally exhibit high susceptibility to tigecycline, with a sensitivity rate of up to 98.0% across various strains. However, this susceptibility declines in MDR (93.1%) or pan-resistant strains (87.8%) (Pfaller et al., 2018). It’s noted that combination therapies involving tigecycline and other antibiotics have been shown to yield superior clinical outcomes (Korczak et al., 2024). Common combinations include tigecycline-imipenem, tigecycline-polymyxin, and tigecycline-aminoglycoside, all of which demonstrate synergistic effects *in vitro* and exhibit potent antibacterial activity against both *E. coli* and *K. pneumoniae* (Ni et al., 2016; Dundar et al., 2018; Korczak et al., 2024). However, the combination of tigecycline and meropenem has shown antagonistic effects specifically in the treatment of KPC-producing *K. pneumoniae* (Bi et al., 2019).

Tigecycline has been approved by the FDA for the treatment of skin and soft tissue infections, HAP or VAP, and IAIs. Clinically, it demonstrates potent activity against multiple CRE phenotypes and is therefore considered as a last-line option for severe MDR or CRE infections (Liu et al., 2019). Future efforts should focus on optimizing combination therapies, strengthening antimicrobial resistance surveillance, and implementing individualized dosing strategies to address increasingly complex resistance mechanisms.

#### 3.2.5 Eravacycline

Eravacycline is a novel fluorocycline antibiotic with broad-spectrum antibacterial activity against Gram-negative bacteria. While structurally analogous to tigecycline, it features targeted modifications that enhance its binding to the bacterial 30s ribosomal subunit to exert bactericidal effects (Zhanel et al., 2016). Eravacycline’s antibacterial activity is independent of β-lactamase type, and it overcomes traditional tetracycline resistance mechanisms, resulting in significantly higher antibacterial activity against CRE compared to tigecycline (mean MIC ratio = 0.76) (Clark et al., 2020).

In two phase III randomized trials for complicated IAIs, eravacycline demonstrated clinical cure rates non-inferior to those of ertapenem (86.8% vs. 87.6%) and meropenem (90.8% vs. 91.2%) (Solomkin et al., 2017; Solomkin et al., 2019). In ESBL-producing Enterobacterales infections, eravacycline achieved a higher clinical cure rate (14/16, 87.5%) compared to meropenem (11/13, 84.6%) (Solomkin et al., 2019). Additionally, eravacycline has been shown to exert synergistic effects when combined with other antibiotics. It exhibits potent activity against *E. coli* and *K. pneumoniae* when combined with polymyxin B, while also showing enhanced antibacterial efficacy when used in conjunction with ceftazidime or imipenem (Li et al., 2022). In adverse effects, eravacycline shows a lower incidence compared to other tetracyclines, such as less gastrointestinal reactions (e.g., nausea, vomiting, diarrhea), and reveals favorable outcomes in preventing relapse and alleviating clinical symptoms (Solomkin et al., 2019; Alosaimy et al., 2020).

Eravacycline has been demonstrated by multiple studies to be effective against various CRE phenotypes and is currently approved by both the FDA and EMA for complicated IAIs. However, its use in CRE BSIs and UTIs is limited due to its suboptimal concentrations in serum and urine (Tamma et al., 2023a). Currently, high-level clinical evidence supporting its application in CRE bacteremia and pneumonia remains lacking, thus necessitating additional RCTs to confirm its efficacy and optimize combination therapeutic strategies.

### 3.3 New drugs in the pipeline

#### 3.3.1 Apramycin

Apramycin, an aminoglycoside antibiotic primarily utilized in veterinary practice, evades the activity of most aminoglycoside-modifying enzymes and 16S rRNA methyltransferases (Camelena et al., 2023). Its antibacterial activity against methylated ribosomes is over 100-fold greater than that of other aminoglycosides (Frimodt-Moller et al., 2024).

Recent *in vitro* studies show apramycin exhibits potent antibacterial activity against CRE. Among clinical CRE isolates, the susceptibility rate to apramycin reaches 70.8%, which is significantly higher than that of commonly used aminoglycosides (gentamicin: 47.2%; tobramycin: 34.7%) (Smith and Kirby, 2016). In a study of 470 MDR Gram-negative bacteria (including CRE), apramycin demonstrated a low MIC_90_ values (8 μg/mL) against Enterobacterales, with apparent activity against nearly all CRE strains, *A. baumannii*, and *P*. *aeruginosa* (Gysin et al., 2022). Additionally, among 6973 CRE bloodstream isolates, the resistance rate to apramycin was only 2.1%, much lower than that of colistin (46.4%) (Frimodt-Moller et al., 2024).

Although still in phase I clinical trials, apramycin shows considerable therapeutic potential for the treatment of CRE. Future research efforts should focus on developing apramycin derivatives to counteract potential resistance and evaluating its safety profile for clinical application.

#### 3.3.2 Sitafloxacin

Sitafloxacin, a fourth-generation fluoroquinolone antibiotic, interferes with DNA replication by inhibiting bacterial DNA gyrase and topoisomerase IV (Akasaka et al., 1998). It demonstrates broad-spectrum antibacterial activity *in vitro*, with notable potency against MDR strains such as quinolone-resistant or ESBL-producing Enterobacterales (Guo et al., 2022). Studies indicate that sitafloxacin has superior *in vitro* activity against CRE-associated strains compared to some traditional fluoroquinolones (Anderson, 2008).

Clinical studies of sitafloxacin in CRE infections are relatively limited. In a simulated human urinary pharmacokinetic model, sitafloxacin exhibited significant bactericidal activity against MDR-CRE strains (e.g., carbapenem-resistant *E. coli*) (Guo et al., 2022). It predicts that a dosing regimen of 100 mg/day may achieve a 90% target attainment rate for CRE isolates with MIC values ≤ 8 mg/L (Guo et al., 2022). The common adverse events of sitafloxacin include gastrointestinal disturbances (17.2%) and laboratory abnormalities (16.2%, such as elevated liver enzymes) (Anderson, 2008).

Sitafloxacin has been approved in Japan for the treatment of respiratory tract infections and UTIs caused by susceptible pathogens, and was approved in China in 2020 (Keating, 2011). Sitafloxacin is primarily indicated for the treatment of infections caused by NDM-producing CRE. However, the global use of sitafloxacin is limited by the lack of large-scale clinical data in CRE infections, as well as its potential risk of phototoxicity among Caucasians (Anderson, 2008). Future researches are needed to further explore the role of sitafloxacin in CRE infections, particularly considering its clinical utility in regions with high antimicrobial resistance rates.

#### 3.3.3 Cefepime-taniborbactam

Cefepime-taniborbactam exhibits potent activity against CRE strains producing both SBLs and MBLs. Taniborbactam exhibits potent activity against most carbapenemases but demonstrates weaker activity against IMP-type enzymes (Zhanel et al., 2024). By binging to β-lactamases to inhibit carbapenemase-mediated hydrolysis of cefepime, taniborbactam restores the bactericidal activity of cefepime against CRE (Roach et al., 2021).

Cefepime-taniborbactam has demonstrated significant efficacy in both *in vitro* and clinical trials. At a concentration of ≤16 μg/mL, cefepime-taniborbactam inhibited 99.5% of Enterobacterales strains, over 97% of MDR strains, over 89% of meropenem-resistant, showing high *in vitro* activity against various CRE phenotypes (Karlowsky et al., 2024). The phase III trial CERTAIN-1 confirmed a clinical success rate of 88.9% (8/9 cases) in patients with complicated UTIs caused by carbapenemase-producing strains (Moeck et al., 2024).

Cefepime-taniborbactam is currently in clinical development. In a phase III randomized double-blind trial for hospitalized adults with complicated UTIs, including acute pyelonephritis, it demonstrated superior efficacy compared to meropenem, with no major adverse events reported (Wagenlehner et al., 2024). Although available data remain limited, future studies should focus on collecting additional clinical evidence to explore its potential applications in other types of infections.

#### 3.3.4 Cefepime-zidebactam

Cefepime-zidebactam is a combination of the β-lactam antibiotic cefepime and the β-lactamase inhibitor zidebactam. Zidebactam can inhibit the β-lactamase activity in Gram-negative bacteria and bind to bacterial PBP2, thereby enhancing the disruptive effect of cefepime on the cell wall (Sader et al., 2017b).

*In vitro* studies have demonstrated the potent inhibitory effect of this dual mechanism against Enterobacterales. The combination exhibits very low MIC values against Enterobacterales. At a 1:1 ratio, the MIC_90_ of cefepime-zidebactam ranges from ≤0.03 to 0.5 μg/mL, inhibiting 99.3% of CRE strains at a concentration of ≤8 μg/mL (Sader et al., 2017a). Compared to cefepime monotherapy, the addition of zidebactam significantly enhanced antibacterial activity, reducing MIC values by more than 2-fold (Sader et al., 2017a). A test involving 204 NDM-producing Enterobacterales isolates showed that zidebactam reduced the cefepime’s MIC in 71.1% (145/204) of the strains (Liu X. et al., 2025). *In vivo* studies also support the therapeutic potential of cefepime-zidebactam against SBLs-producing *K. pneumoniae*. A study using a neutropenic murine pneumonia model to simulate human plasma and epithelial lining fluid (ELF) drug exposure levels, confirmed that the combination effectively reduced bacterial load, indicating meaningful efficacy in infection-relevant physiological conditions. (Lasko et al., 2021).

Animal model studies suggest good tolerability for cefepime-zidebactam, but its safety in humans has not been fully evaluated through clinical trials (Lasko et al., 2021). Cefepime-zidebactam remains in clinical development, with ongoing research focused on defining its therapeutic role against MDR pathogens, including CRE.

#### 3.3.5 Meropenem-xeruborbactam

Xeruborbactam is a broad-spectrum β-lactamase inhibitor that effectively inhibits SBLs and MBLs. Its unique cyclic boronic acid structure enables covalent enzyme binding and inactivation, significantly enhancing the antibacterial activity of carbapenems (Sun et al., 2022). In addition, xeruborbactam itself exhibits direct antibacterial activity against CRE, with MIC_50_/MIC_90_ values of 16/32 μg/mL (Sun et al., 2022).

*In vitro* experiments have demonstrated that xeruborbactam has favorable antibacterial activity. In a study of 115 clinical CRE isolates, the addition of 8 μg/mL xeruborbactam reduced the MIC_90_ of meropenem from 128 mg/L to 0.25 mg/L (Ooi et al., 2021). In a murine infection model, subcutaneous infection of meropenem-272 (a xeruborbactam analog) effectively eradicated NDM/IMP/VIM-producing Enterobacterales, which confirming its *in vivo* efficacy (Ooi et al., 2021). Meropenem-xeruborbactam overcomes the limitations of meropenem monotherapy and meropenem-vaborbactam combination therapy against MBLs-producing strains (Le Terrier et al., 2025). In a *in vitro* analysis of 300 clinical CRE isolates, meropenem-xeruborbactam demonstrated superior susceptibility (100%) compared to other therapeutic combinations tested against this collection (Sanchez-Pena et al., 2025).

Current research on meropenem-xeruborbactam suggests it has high therapeutic potential against CRE and may emerge as a preferred treatment option, especially for infections caused by KPC and MBL producers (Sanchez-Pena et al., 2025). However, present investigations are mainly at the pre-clinical stage, and phase III trials are urgently needed to confirm its efficacy in patients with CRE infections.

### 3.4 Other scenarios

#### 3.4.1 Mecillinam

Mecillinam is a broad-spectrum semi-synthetic penicillin that acts through high-affinity specific binding to PBP2 (Neu, 1985). In the treatment of CRE infections, its combination with carbapenems or other β-lactams enhances cell wall disruption in resistant Enterobacterales, inducing bacterial spherical transformation and eventual lysis (Kramer et al., 1983).

Mecillinam demonstrates limited efficacy as monotherapy against CRE, primarily due to the frequent carriage of carbapenemases or MBLs, which may lead to hydrolysis and inactivation of mecillinam (Neu, 1985). However, when combined with ampicillin, carbenicillin, or cephalosporins, its bactericidal activity against CER is significantly enhanced (Kramer et al., 1983; Neu, 1983). In UTI-associated CRE infections (particularly *E. coli* and *K. pneumoniae*), the combination of mecillinam with amoxicillin/clavulanate can effectively overcome such resistance (Birgy et al., 2017). Preclinical studies confirm that mecillinam combined with meropenem or other carbapenems may restore the activity of carbapenem against certain CRE strains (Jin et al., 2018).

Currently, mecillinam is recommended primarily for uncomplicated UTIs caused by susceptible Enterobacterales (e.g., *E. coli*, *Klebsiella species*) (Emeraud et al., 2022), especially those with OXA-48 and NDM phenotypes. Although reported resistance rates remain relatively low (59/394, 15%) (Milleville et al., 2025), monotherapy is inadequate for severe CRE infections, such as bacteremia or pneumonia. Future clinical research should evaluate combination regimens incorporating mecillinam for severe CRE infections.

#### 3.4.2 Cefepime-enmetazobactam

Cefepime-enmetazobactam is a novel β-lactam/β-lactamase inhibitor combination. Enmetazobactam can inhibit Ambler class A ESBLs but is ineffective against MBLs (Bhowmick et al., 2025; Bonnin et al., 2025). Its mechanism involves enmetazobactam binding to β-lactamases, thereby restoring the antibacterial activity of cefepime against ESBLs-producing bacteria (Bhowmick et al., 2025).

The *in vitro* tests have confirmed that enmetazobactam can restore the activity of ceftazidime against OXA-48-producing Enterobacterales, effectively reducing the MIC (MIC_90_ decreased from >16 mg/L to 1 mg/L) (Bonnin et al., 2025). In a phase III trial involving adults with complicated UTI, cefepime-enmetazobactam demonstrated superiority over piperacillin-tazobactam in both clinical cure rates and microbiological eradication (Kaye et al., 2022). However, data for CRE pneumonia are lacking, emphasizing the need for additional clinical investigations to validate efficacy in this indication. The incidence of adverse events to cefepime-enmetazobactam treatment is relatively high (258/516, 50%), but the rate of treatment discontinuation due to adverse events is low (1.7% in the cefepime-enmetazobactam group vs. 0.8% in the piperacillin group), without reported severe hepatotoxicity or nephrotoxicity (Kaye et al., 2022; Darlow et al., 2025). *In vitro* studies have revealed that cefepime-enmetazobactam retains potent antibacterial activity against ESBL-producing and AmpC-producing strains, and exhibits a high resistance rate (87.5%) against MBL-producing strains (Falagas et al., 2025).

Cefepime-enmetazobactam has been approved by the FDA for the treatment of complicated UTIs in adults. The EMA has also approved it for complicated UTIs, HAP/VAP, and associated bacteremia. However, its ineffectiveness against KPC and MBLs limits its broad-spectrum application. Future focus should be on generating clinical data for CRE pneumonia and exploring combination therapy strategies.

#### 3.4.3 Tavaborole combined with meropenem

Tavaborole is an FDA-approved antifungal agent used for the treatment of onychomycosis (Gupta and Versteeg, 2016), which exerts its effect by potently inhibiting fungal leucyl-tRNA synthetase (Rock et al., 2007). Recent studies indicated that tavaborole may serve as a potential broad-spectrum inhibitor of both SBLs and MBLs (Zhang et al., 2025). When combined with meropenem, tavaborole has been shown to enhance meropenem’s antibacterial activity, as demonstrated in both *in vitro* assays and animal infection models (Liu M. et al., 2025; Zhang et al., 2025). These fingdings highlight tavaborole as a promising candidate for repurposing and provide a novel therapeutic approach for the management of CRE infections.

#### 3.4.4 Carvacrol combined with meropenem

Carvacrol, a monoterpenoid phenolic compound, has been shown to inhibit biofilm formation in CRE (Raei et al., 2017). Both *in vitro* and animal studies have demonstrated its antibacterial activity against CRKP (de Souza et al., 2021). An *in vitro* investigation of carvacrol combined with meropenem revealed synergistic effects against tested CRKP strains (8/25), whereas neither agent alone exhibited bactericidal activity against these isolates (Kose, 2022). Collectively, these findings suggest that carvacrol may represent a potential adjunctive therapy when used in combination with conventional antibiotics for the treatment of CRE infections. However, well-designed RCTs are required to validate the clinical efficacy and safety of such combination strategies (Table 1).

**TABLE 1:** Summary of antibiotics in the pipeline and their spectrum of activities.

Name	Development/Regulatory status	Antibiotic class	Efficacy signal	Indication/Usage
*First-line therapy*				
Cefiderocol	FDA/EMA/CDA approved	Cephalosporins	Non-inferior to BAT for CRE infections	UTIs
Ceftazidime-avibactam	FDA/EMA/CDA approved	β-lactam/β-lactamase	Superior to traditional regimens for KPC-producing strains	UTIs, IAIs, HAP
Meropenem-vaborbactam	FDA/EMA/CDA approved	β-lactam/β-lactamase	Superior to BAT for KPC-producing CRE infections	UTIs, IAIs, HAP, VAP
Aztreonam-avibactam	FDA/EMA/CDA approved	β-lactam/β-lactamase	Effective against MBL-producing strains	IAIs
Imipenem-cilastatin-relebactam	FDA/EMA/CDA approved	β-lactam/β-lactamase	Effective against imipenem-non-susceptible gram-negative infections	UTIs, IAIs
*Older agents recommended in guidelines*				
Fosfomycin	FDA/EMA/CDA approved	​	Oral formulation effective for uncomplicated UTIs caused by MDR *E. coli*	UTIs
Polymyxins	FDA/EMA/CDA approved	​	Often used in combination, but significant nephrotoxicity	BSIs, IAIs, UTIs, VAP
Aminoglycosides	FDA/EMA/CDA approved	​	Effective for susceptible UTIs, but high resistance and toxicity	UTIs
Tigecycline	FDA/EMA/CDA approved	Tetracyclines	Effective for complicated IAI, but low serum levels preclude monotherapy for bacteremia	Skin and soft tissue infections, IAIs
Eravacycline	FDA/EMA/CDA approved	Tetracyclines	Non-inferior to ertapenem, with activity against some tetracycline-resistant strains	IAIs
*Clinical research phase*				
Sitafloxacin	Japan/CDA approved	Fluoroquinolone	Effective for UTIs caused by susceptible pathogens, but ineffective for caucasians	UTIs
Apramycin	Phase I	Aminoglycosides	Preclinical data shows efficacy against MDR gram-negatives	BSIs
Cefepime-taniborbactam	Phase III	β-lactam/β-lactamase	Phase III met primary endpoint in complicated UTI, superior to meropenem	UTIs
Cefepime-zidebactam	Phase III	β-lactam/β-lactamase	Early data shows promising activity against MDR *P. aeruginosa* and CRE.	Pneumonia, BSIs
Meropenem-xeruborbactam	Phase III	β-lactam/β-lactamase	Preclinical data shows potent inhibition of carbapenemases	UTIs, IAIs, HAP, VAP

## 4 Discussion

The treatment of CRE infections represents a critical and evolving challenge in infectious diseases. The primary rationale for investigating combination regimens, particularly those pairing a β-lactam with a novel inhibitor, is to overcome specific enzymatic resistance (e.g., KPC, NDM, OXA-48) while leveraging potential synergistic killing and reducing the risk of emergent resistance. However, the literature presents conflicting evidence on the superiority of combination therapy over monotherapy with novel β-lactam/β-lactamase inhibitor combinations. For instance, while some studies suggest a benefit for combinations like ceftazidime-avibactam, other analyses find no significant mortality difference compared to effective monotherapy (Torres et al., 2018). These discrepancies may stem from differences in study populations (e.g., severity of illness, proportion of ICU patients, source of infection), local carbapenemase epidemiology, dosing strategies (prolonged vs. short infusions), and the choice of comparator, which is often confounded by indication in non-randomized studies.

Beyond efficacy, significant practical barriers hinder the real-world use of even the most potent regimens. While traditional antibiotics like polymyxins, tigecycline, and aminoglycosides were previously considered first-line options, their utility is increasingly constrained by rising resistance rates and significant toxicity concerns. Although novel β-lactam/β-lactamase inhibitor combinations have expanded therapeutic possibilities, their clinical application is hindered by multiple constraints, including limited availability, uneven global distribution, and variable efficacy across resistance phenotypes (Zasowski et al., 2015; Rodriguez-Bano et al., 2018; Cabello et al., 2024). Furthermore, the frequent emergence of extensively drug-resistant (EDR) and PDR CRE phenotypes exacerbates therapeutic challenges (Thaden et al., 2017; Song et al., 2020). Co-resistance to carbapenems, other β-lactams, aminoglycosides, and fluoroquinolones further contributes to highly complex MDR profiles that are exceptionally difficult to manage (Durante-Mangoni et al., 2019; Song et al., 2020).

To address antimicrobial resistance, researchers have proposed five strategic scientific directions: developing narrow-spectrum antibiotics from natural products targeting specific bacteria; utilizing AI for antibiotic screening and design; exploring novel synergistic drug combinations; reducing antibiotic use through immunomodulation; and enhancing rapid pathogen detection technologies (Dance, 2024). Among these, combination therapies have shown preliminary success against carbapenem-resistant *A. baumannii* and CRKP (Kose, 2022; Gadar et al., 2023). It is noteworthy that some countries lack many of the recommended antibiotics even though they report them in their studies, since automated equipment shows general results from identification cards even though those antimicrobials are not actually used. Advances in rapid molecular diagnostics now enable precision combination therapy for CRE infections. Recent studies demonstrate that multiplex real-time polymerase chain reaction can complete carbapenemase gene genotyping within hours (Pancotto et al., 2018), providing crucial evidence for clinical decision-making. A multi-center study involving eight institutions in the New York-New Jersey area confirmed that regional data sharing significantly reduced mortality among patients with CRE bacteremia (Satlin et al., 2022), underscoring the value of coordinated antimicrobial resistance surveillance networks. Meanwhile, the emergence of novel therapeutic modalities such as phage therapy offers innovative approaches for CRE treatment (Rotman et al., 2024).

The limitations of this review must be acknowledged. Our focus has been on traditional and newer antimicrobial chemotherapies, intentionally omitting a detailed discussion of emerging biological therapies such as phage therapy or immunomodulatory agents, which represent a distinct and promising frontier. Furthermore, as a narrative review, our synthesis is inherently limited by the methodological constraints of the format, including potential selection bias in the cited literature and the inability to perform a quantitative meta-analysis of the evidence presented.

In conclusion, the management of CRE infections is transitioning from an era of desperation to one of strategic, mechanism-based choice. Future directions should include conducting well-designed RCTs to inform clinical guidelines, promoting widespread implementation of rapid molecular diagnostic tools for early detection, and fostering the development of novel antimicrobials and combination treatment strategies to more effectively address the global threat posed by CRE.

## Footnotes

1
https://www.cdc.gov/cre/about/
